# Challenges for Hybrid Water Electrolysis to Replace the Oxygen Evolution Reaction on an Industrial Scale

**DOI:** 10.1002/gch2.202200242

**Published:** 2023-05-11

**Authors:** Till Kahlstorf, J. Niklas Hausmann, Tobias Sontheimer, Prashanth W. Menezes

**Affiliations:** ^1^ Material Chemistry Group for Thin Film Catalysis–CatLab Helmholtz‐Zentrum Berlin für Materialien und Energie Albert‐Einstein‐Str. 15 12489 Berlin Germany; ^2^ Strategy Department of Energy and Information Helmholtz‐Zentrum Berlin für Materialien und Energie Hahn‐Meitner‐Platz 1 14109 Berlin Germany; ^3^ Department of Chemistry Technische Universität Berlin Straße des 17 Juni 135, Sekr. C2 10623 Berlin Germany

**Keywords:** co‐electrolysis, electrooxidation of biomass, hybrid water electrolysis, hybrid water splitting, industrial scale, techno‐economic analysis, value‐added organic oxidation reaction

## Abstract

To enable a future society based on sun and wind energy, transforming electricity into chemical energy in the form of fuels is crucial. This transformation can be achieved in an electrolyzer performing water splitting, where at the anode, water is oxidized to oxygen—oxygen evolution reaction (OER)—to produce protons and electrons that can be combined at the cathode to form hydrogen—hydrogen evolution reaction (HER). While hydrogen is a desired fuel, the obtained oxygen has no economic value. A techno‐economically more suitable alternative is hybrid water electrolysis, where value‐added oxidation reactions of abundant organic feedstocks replace the OER. However, tremendous challenges remain for the industrial‐scale application of hybrid water electrolysis. Herein, these challenges, including the higher kinetic overpotentials of organic oxidation reactions compared to the OER, the small feedstock availably and product demand of these processes compared to the HER (and carbon dioxide reduction), additional purifications costs, and electrocatalytic challenges to meet the industrially required activities, selectivities, and especially long‐term stabilities are critically discussed. It is anticipated that this perspective helps the academic research community to identify industrially relevant research questions concerning hybrid water electrolysis.

## Graphical abstract

Hybrid water electrolysis has emerged as a promising field for the coproduction of hydrogen and value‐added chemicals. Herein, a short overview of the reactions investigated and the challenges to be solved to accelerate developments toward industrial‐scale applications is given.

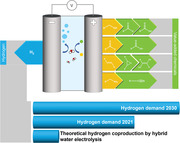

## 1 Introduction

Currently, society's energy demand is mostly supplied by chemical energy in the form of fossil fuels that is subsequently converted into its kinetical, electric, or thermal forms.^[^ ^1^, ^2^, ^3^ ^]^ This conversion is performed by burning the chemical energy carriers where ambient oxygen is reduced, and the fuels are oxidized, forming water and carbon dioxide.^[^ ^1^ ^]^ On the contrary, in a future carbon‐neutral society, wind and solar energy plants will produce electricity that must be converted into chemical, kinetical, and thermal energy. While the transformation into kinetical and thermal energy can be performed already with high efficiency and at comparably low costs, the conversion into chemical energy is challenging.^[^ ^4^ ^]^ One possible way is the storage in batteries, which is highly efficient, but requires the construction of resource‐ and cost‐intensive batteries.^[^ ^5^, ^6^ ^]^ Alternatively, the fuel cycle can be closed by reversing the burning of fuels, meaning that the oxygen in water or carbon dioxide is oxidized to ambient dioxygen again (oxidation states are in blue)^[^ ^4^ ^]^

(1) $$\begin{array}{c} 2H_{2}O^{-\mathrm{II}}\to 4H^{+}+4e^{-}+O_{2}^{0} \end{array}$$

(2) $$\begin{array}{c} {\mathrm{CO}}_{2}^{-\mathrm{II}}\to \left[C^{4+}\right]+4e^{-}+O_{2}^{0} \end{array}$$

These processes are thermodynamically up‐hill and thus require an external driving force, such as electricity from wind and solar power plants.^[^ ^4^, ^6^ ^]^ Therefore, these reactions must be performed in an electrolyzer, where they take place in the oxidizing environment of the anode. While the first reaction is well established and called oxygen evolution reaction (OER), the second has not been successfully applied to the best of our knowledge. This is caused by the energetically extremely demanding [C^4+^] species that would theoretically form at the anode and must be transported to the cathode, where it could be reduced to, e.g., elemental carbon. In contrast, in the first reaction, protons are formed, which can be stabilized in aqueous media sufficiently well and transported to the reducing compartment, i.e., the cathode. At the cathode, these protons, together with the electrons taken from the O^−II^ species, can be used to form fuels. In the simplest case, both are merely combined to form H^0^_2_; however, they can also be combined with other small molecules, such as carbon dioxide, to form carbon‐based fuels such as alcohols or hydrocarbons.^[^ ^4^, ^6^, ^7^ ^]^

The advantage of the OER is that water is sufficiently abundant and cheap to supply enough electrons for all desired fuel‐forming cathodic reactions.^[^ ^8^ ^]^ However, the disadvantages of the OER are that it is thermodynamically and kinetically demanding to oxidize water and that the produced oxygen has no economic value. Furthermore, when hydrogen and oxygen are produced simultaneously, membranes or diaphragms must be used to separate the two gases to achieve a high hydrogen purity.^[^ ^9^ ^]^ To overcome these disadvantages, researchers have explored alternative oxidation reactions to supply electrons and protons.^[^ ^10^, ^11^, ^12^, ^13^, ^14^, ^15^, ^16^, ^17^, ^18^, ^19^, ^20^, ^21^, ^22^, ^23^, ^24^, ^25^ ^]^ These alternative approaches use organic substrates that are oxidized into value‐added non‐gaseous products, e.g., the oxidation of glycerol to lactic acid or the oxidation of biomass‐derived 5‐hydroxymethylfurfural (HMF) to 2,5‐furandicarboxylic acid (FDCA)—a potential precursor for future green polymers. This replacement of the OER in an electrolyzer is called hybrid water electrolysis. Such an approach promises several advantages over traditional water splitting: The applied organic substrates have substantially lower oxidation potentials than water, and thus the cell voltage of the electrolyzer can be much lower, which can significantly increase the amount of hydrogen obtained per invested electricity—the main cost factor of hydrogen production together with the electrolyzer price.^[^ ^8^, ^9^ ^]^ Therefore, hybrid water electrolysis could lead to a substantial cost reduction of electrocatalytic hydrogen production and enable the simultaneous production of an anodic value‐added organic product. In addition, as the oxidation is driven electrochemically, the frequently required use of stoichiometric oxidation chemicals and harsh reaction conditions can be avoided. Another advantage of applying organic oxidation reactions (OOR) instead of the OER is the avoidance of explosive H_2_/O_2_ mixtures caused by gas diffusion from one electrode compartment to the other. Moreover, the membrane's lifetime could also be improved as no reactive oxygen species are formed during the process. Excellent and comprehensive reviews on hybrid water electrolysis have been published already, covering electrolyzer design, catalyst development, substrate variety, and mechanistic insights.^[^ ^10^, ^11^, ^12^, ^13^, ^14^, ^15^, ^16^, ^17^, ^18^, ^19^, ^20^, ^21^, ^22^, ^23^, ^24^, ^25^ ^]^

In contrast to these previous reviews, in this perspective, we focus on the challenges of hybrid water electrolysis and answer the following questions:

- Why are the overpotentials required for organic substrate oxidation usually substantially larger than the ones required for the OER?
- What is the current and future demand of the organic compounds compared to the one of hydrogen?
- How does the feedstock availability compare to the electron demand of hydrogen production?
- To what extent can the OER be replaced, considering the large hydrogen demand?
- What challenges is the application of hybrid water electrolysis facing, regarding the catalyst's selectivity, stability, and activity?

## 2 Overview on Potential Substrates

**Figure 1:**
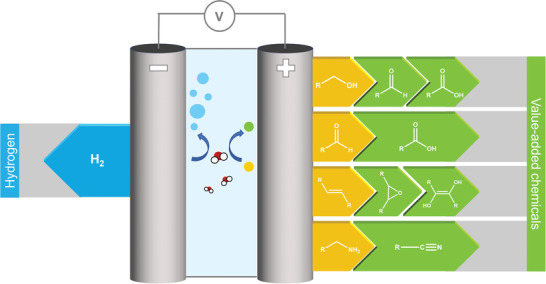
Schematic illustration of hybrid water electrolysis systems showing cathodic hydrogen production on the left and anodic substrates (yellow) and their value‐added products (green) on the right.

During the last decade, various organic compounds have been tested as potential feedstocks for hybrid water electrolysis systems to replace the anodic OER. In this regard, the most promising and best‐studied classes of compounds are illustrated in **Figure** **1**, including alcohols, aldehydes/ketones, alkenes, and amines. In addition, there are, of course, a variety of other reactions that have been demonstrated to replace the OER. For instance, the electrochemical oxidation of sulfides to sulfoxides could be useful in pharmaceutical synthesis.^[^ ^13^ ^]^ However, this is a small‐scale process compared to the value‐added products discussed herein. Other efforts not covered are oxidation reactions that yield no valuable products, e.g., when the feedstock is a sacrificial agent or a pollutant is degraded. A prominent example is urea, which can be oxidized to form N_2_, H_2_, and CO_2_ in a 6e^−^ process. While the reaction requires less electricity than the OER and provides additional H_2_, it produces undesirable CO_2_ and does not provide another value‐added chemical; in contrast, it consumes urea, a valuable chemical, even though it might be present as an impurity in wastewater streams.

**Table** **1** provides a brief overview of the organic oxidation reactions that are claimed to be value‐added and have been explored recently. These reactions use water as the oxygen source and can be classified by their functional groups and respective oxidation reactions. In this regard, the anodic partial oxidation of small alcohols yields aldehydes/ketones in a 2e^−^ process or the corresponding carboxylic acids in a 4e^−^ process. Similarly, aldehydes are oxidized, forming carboxylic acids. Furthermore, the oxidative coupling of two alcohols enables the production of esters. For example, coupling two ethanol molecules can yield ethyl acetate, a chemical with a wide range of industrial applications. Oxidative ring openings can also be accomplished, e.g., producing adipic acid, an essential precursor for the production of nylon, from cyclohexanone/cyclohexanol.^[^ ^26^ ^]^ Larger biomass‐derived compounds contain multiple reactive oxygen sites, such as 5‐hydroxymethylfurfural (HMF) or glycerol. Thus, various products can be produced, depending on applied reaction conditions and catalyst, e.g., glycerol oxidation has at least ten conceivable, commercially relevant oxidation products.^[^ ^27^, ^28^ ^]^ In this regard, the glycol oxidation mechanism network is complex and involves potential C—C bond cleavages producing smaller molecules such as formic acid, lactic acid, or nonvaluable carbon dioxide. Moreover, alkenes are promising potential feedstocks as they enable the oxidative synthesis of epoxides, diols, and higher oxidized species. The last class of substrates included herein are amines, which can either be oxidized to nitriles via dehydrogenation or coupled with each other to form diazo compounds. Notably, these hybrid water electrolysis processes are only viable on an industrial scale if there is a future market for the products, which is discussed in Section 3.2.

**Table 1:** Overview of organic oxidation reactions yielding value‐added chemicals in hybrid water electrolysis. Thermodynamic overpotentials for the anodic half‐reactions are taken from a study by Na et al.^[^ ^29^ ^]^ These processes can be coupled with the HER, which has a thermodynamic overpotential of 0 V versus standard hydrogen electrode (*V*_SHE_). Please note that the potentials of all these processes are pH dependent. When acids or bases are formed, the pH dependencies might differ from those of the HER and OER. In all other cases, where the same number of protons and electrons is added or removed during the reaction, the pH dependency is the same (59 mV pH^−1^ for one proton per electron) as for the HER and OER. Thus, the pH will not change the potential difference between the HER and the organic oxidation reaction.^[^ ^30^ ^]^ Comprehensive reviews of the listed reactions have been published already.^[^ ^12^, ^13^, ^15^, ^16^, ^31^ ^]^

Substrate			Product		Potential [V_SHE_]
Water	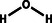	**→**	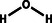	Oxygen	1.23
		**→**	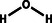	Hydrogen peroxide	1.78
Methanol	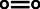	**→**	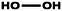	Formaldehyde	
		**→**	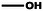	Formic acid	−0.258
Methane	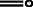	**→**	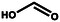	Methanol	
Ethanol	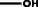	**→**	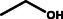	Ethyl acetate	−0.208
		**→**	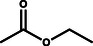	Acetic acid	−0.334
		**→**	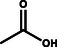	Acetaldehyde	0.193
Isopropanol	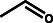	**→**	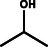	Acetone	0.054
Ethylene	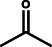	**→**	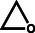	Ethylene oxide	
		**→**	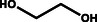	Ethylene glycol	
Ethylene glycol	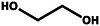	**→**	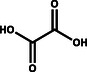	Oxalic acid	−0.455
		**→**	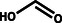	Formic acid	
		**→**	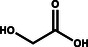	Glycolic acid	−0.334
1,2‐Propanediol	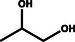	**→**	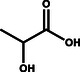	Lactic acid	
1,3‐Propanediol	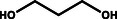	**→**	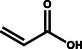	Acrylic acid	
HMF	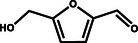	**→**	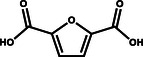	FDCA	−0.78
		**→**	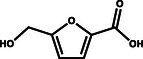	HMFCA	
Furfural	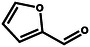	**→**	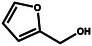	2‐Furoic acid	−1.27
Furfuryl alcohol	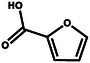	**→**			−0.515
Benzyl alcohol	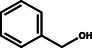	**→**	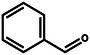	Benzaldehyde	−0.334
		**→**	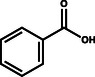	Benzoic acid	0.193
Glycerol	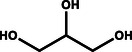	**→**	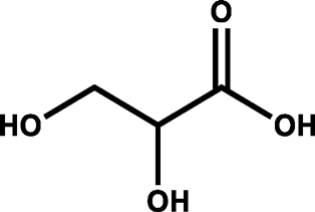	Glyceric acid	
		**→**	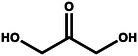	Dihydroxyacetone	
		**→**	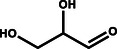	Glyceraldehyde	
		**→**	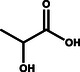	Lactic acid	0.041
		**→**	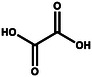	Oxalic acid	
		**→**	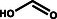	Formic acid	
Cyclohexanone	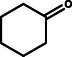	**→**	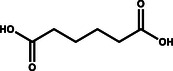	Adipic acid	
Cyclohexanol	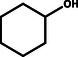	**→**			
Propylamine	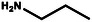	**→**	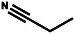	Propionitrile	
Benzylamine	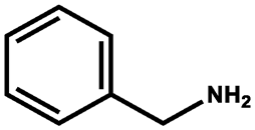	**→**	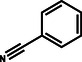	Benzonitrile	

## 3 Challenges of Hybrid Water Electrolysis

### 3.1 Organic Oxidation Reactions Have Enormous Overpotentials Compared to the OER

Table 1 shows that the thermodynamic oxidation potentials of the anodic organic oxidation reactions (<0.5 V_SHE_) in hybrid water splitting are usually substantially lower than the one of the OER (1.23 V_SHE_). Thus, these substrates are easier to oxidize than water, which makes sense, as, in the OER, electrons are removed from oxygen—the element with the second highest electronegativity of the periodic table; while, in the anodic organic reactions, electrons are removed from carbon with substantially lower electronegativity. Based on this thermodynamic property, hybrid water splitting could potentially be performed at minute cell potentials. As the HER potential is 0 V_SHE_, the minimum required cell potential is equal to the potential of the anodic process. In a few cases, e.g., the oxidation of furfural to furoic acid, the potential of the organic oxidation reaction (−1.27 V_SHE_) is even below the one of the HER (0 V_SHE_), meaning that no externally applied electricity is required. Instead, electricity could even be obtained by the process. However, in real systems, the potentials required for the organic oxidation reaction are usually only slightly below (1.2–1.6 V_SHE_) the ones required for the OER (1.4–1.7 V_SHE_). Thus, the organic oxidation reactions’ kinetic overpotential is tremendously larger than the OER's, almost completely eliminating these oxidation processes' substantial thermodynamic advatages.

In this regard, studies are interesting that compare the OER and various organic oxidation reactions using the same catalyst, which our group and others have done several times.^[^ ^10^, ^11^, ^12^, ^13^, ^14^, ^15^, ^16^, ^17^, ^18^, ^19^, ^20^, ^21^, ^22^, ^23^, ^24^, ^25^ ^]^ Especially a recent study oxidizing 24 different substrates with the same nickel‐iron‐based electrode in alkaline media showed that the potentials required for the reactions were all close to the one thermodynamically required for the OER and independent of the oxidation potential of the organic substrate.^[^ ^32^ ^]^ We observed the same similarity in our studies using different alcohols, aldehydes, and ketones as substrates.^[^ ^20^, ^24^ ^]^ The observation that such similar potentials are required for thermodynamically so different processes could be explained by a shared high energy intermediate. Recent reports have shown that this intermediate is activated (electrophilic) oxygen.^[^ ^33^, ^34^, ^35^ ^]^ This oxygen originates from water bonded to the transition metal in a high oxidation state, e.g., nickel(III) or nickel(IV). The transition metal in the high oxidation state removes electron density from the oxygen, making it electrophilic and highly reactive. This water activation process to electrophilic oxygen is also a reaction step of the OER.^[^ ^33^, ^36^ ^]^ Thus, all processes share the same reaction intermediate. The ideal OER catalysts should require 1.23 V for every of its reaction steps, including the activation of water.^[^ ^37^ ^]^ Therefore, if a decent OER catalyst is applied for the oxidation of organic substrates, it will not be able to drive the process substantially below 1.23 V, independent of the redox potential of the organic substrate. Thus, to overcome the large overpotentials, non‐OER catalysts must be investigated for these processes.

### 3.2 Industrial‐Scale Potential Compared to Hydrogen Production, Economic Considerations, Product Demand, Feedstock Availability, and Purification

The anodic organic oxidation reactions should be performed together with the cathodic HER and potentially carbon dioxide reduction. To decide to which extent these anodic and cathodic reactions can be coupled, the demand for their products and the availability of their feedstock must be compared. Starting with hydrogen, in 2021, the global demand was 95 million tons (main contributors: ≈43% for oil refining, ≈36% for ammonia synthesis, and ≈16% for methanol synthesis).^[^ ^38^ ^]^ Due to the crucial role of hydrogen in the decarbonization of various sectors, its demand is expected to reach 130 Mt per year by 2030 and could reach more than 500 Mt by 2050 according to the International Energy Agency (IEA).^[^ ^38^, ^39^ ^]^ Currently, less than 1% of this hydrogen is produced by electrolysis. Instead, it is made from fossil fuels emitting around 900 Mt of carbon dioxide annually.^[^ ^38^ ^]^ To reach carbon neutrality in the future, most of the hydrogen must be produced via electrolysis.^[^ ^39^ ^]^ Additional to this enormous hydrogen demand, carbon dioxide reduction is a competing cathodic process that also must be provided with electrons and protons from the OER or its alternatives.

To understand how much of the electron demand of the electrocatalytic hydrogen production can be supplied with organic oxidation reactions, we will discuss their potential in the following paragraph. The first aspect is that suitable feedstock must be identified. In this regard, currently, the production of commodity chemicals relies heavily on fossil fuels both in feedstock (petroleum) and in heat generation for industrial processes (mostly thermochemical) causing high carbon emissions with severe environmental consequences.^[^ ^40^ ^]^ However, to be relevant in a future decarbonized society, the substrates considered in hybrid water electrolysis should be derived from sustainable resources such as biomass or otherwise unused waste streams.^[^ ^41^ ^]^ Good examples are glycerol—an industrial‐scale byproduct during biodiesel production—and methane—which is mostly converted to syngas (CO/H_2_) and used in energy‐intensive processes like Fischer–Tropsch and Haber Bosch. However, due to a lack of valorization techniques, much methane is flared off directly at the oil and petroleum plants.^[^ ^42^, ^43^ ^]^ **Table** **2** shows the market prices and yearly availability of the feedstock and the demand for the products of promising anodic oxidation reactions, as well as the electrons provided per formula conversion. It should be noted that for some platform chemicals and their corresponding oxidation products like HMF/FDCA, there is no production on an industrially relevant scale yet, leading to volatile market prices and estimated production values. For the substrates isopropanol and benzyl alcohol, feedstock costs exceed the product price, making them unattractive options. In this regard, we note that an impressive techno‐economical study has been conducted by Na et al., calculating the minimum selling price for various anodic oxidation reactions. They showed that many hybrid water electrolysis systems have the potential to produce value‐added chemicals at the anode in a cost‐competitive manner based on current market prices.^[^ ^29^ ^]^

**Table 2:** Production, scale, and prices for feedstocks and the (value‐added) products of their electrocatalytic oxidation. The data are based on studies by Na et al. and Verma et al. if not stated otherwise.^[^ ^29^, ^41^ ^]^

Feedstock	Yearly production [Mt year^−1^]	Market price^[^ ^44^ ^]^ [$ kg^−1^]	Electrons provided	Product	Yearly production [Mt year^−1^]	Market price^[^ ^44^ ^]^ [$ kg^−1^]
Deionized water	–	0.02–0.10 ^[^ ^11^ ^]^	4	O_2_	2.0	0.02–0.04
			2	H_2_O_2_	2.8	0.36–0.66
Glycerol	4.3 ^[^ ^45^ ^]^	0.68–0.95	8	Formic acid	0.9	0.56–1.19
			4	Glyceraldehyde		2.0–2.11 ^[^ ^45^ ^]^
			2	Dihydroxyacetone	0.004 ^[^ ^11^ ^]^	1.9–2.1 ^[^ ^46^ ^]^
			2	Lactic acid	0.45	1.57–1.87 ^[^ ^45^ ^]^
Ethylene glycol	42.1	0.84–1.15	8	Oxalic acid	0.19	0.94–1.46
			6	Formic acid	0.9	0.56–1.19
			4	Glycolic acid	0.04	1.47–1.90 ^[^ ^47^ ^]^
1,2‐Propandiole	1.36 ^[^ ^25^ ^]^	0.90–1.50	4	Lactic acid	0.45	1.57–1.87
1,3‐Propandiole	0.05	2.20	4	Acrylic acid	0.05	2.25–2.88
HMF		0.78–1.12 ^[^ ^48^ ^]^	6	FDCA	0.5*	0.32–5.80
Furfural		1.17–1.82	2	2‐Furoic acid		≈6.23
Furfuryl alcohol		1.25–1.78	4	2‐Furoic acid		≈6.23
Methanol	110	0.30–0.47	4	Formic acid	0.9	0.56–1.19
			2	Formaldehyde	18 ^[^ ^11^ ^]^	0.37–0.74
Methane	**	0.18–0.22 ^[^ ^49^ ^]^	2	Methanol	110	0.37–0.47
Ethylene	141	0.62–1.21	2	Ethylene oxide	26	1.26–1.60
			2	Ethylene glycol	24.1	0.84–1.15
Ethanol	583	0.42–0.74	4	Ethyl acetate	2.7 ^[^ ^50^ ^]^	0.75–1.07
			4	Acetic acid	10 ^[^ ^50^ ^]^	0.43–0.81
			2	Acetaldehyde	1.7 ^[^ ^50^ ^]^	≈1.00
Isopropanol	2.9	1.08–1.55	2	Acetone	6.4	0.73–1.10
Benzyl alcohol		1.89–3.29	4	Benzoic acid	0.1	1.45–1.85
			2	Benzaldehyde	0.1	1.18–1.57
Cyclohexanone	6.0	1.39–2.40	6	Adipic acid	1.5	1.31–1.57

**Figure 2:**
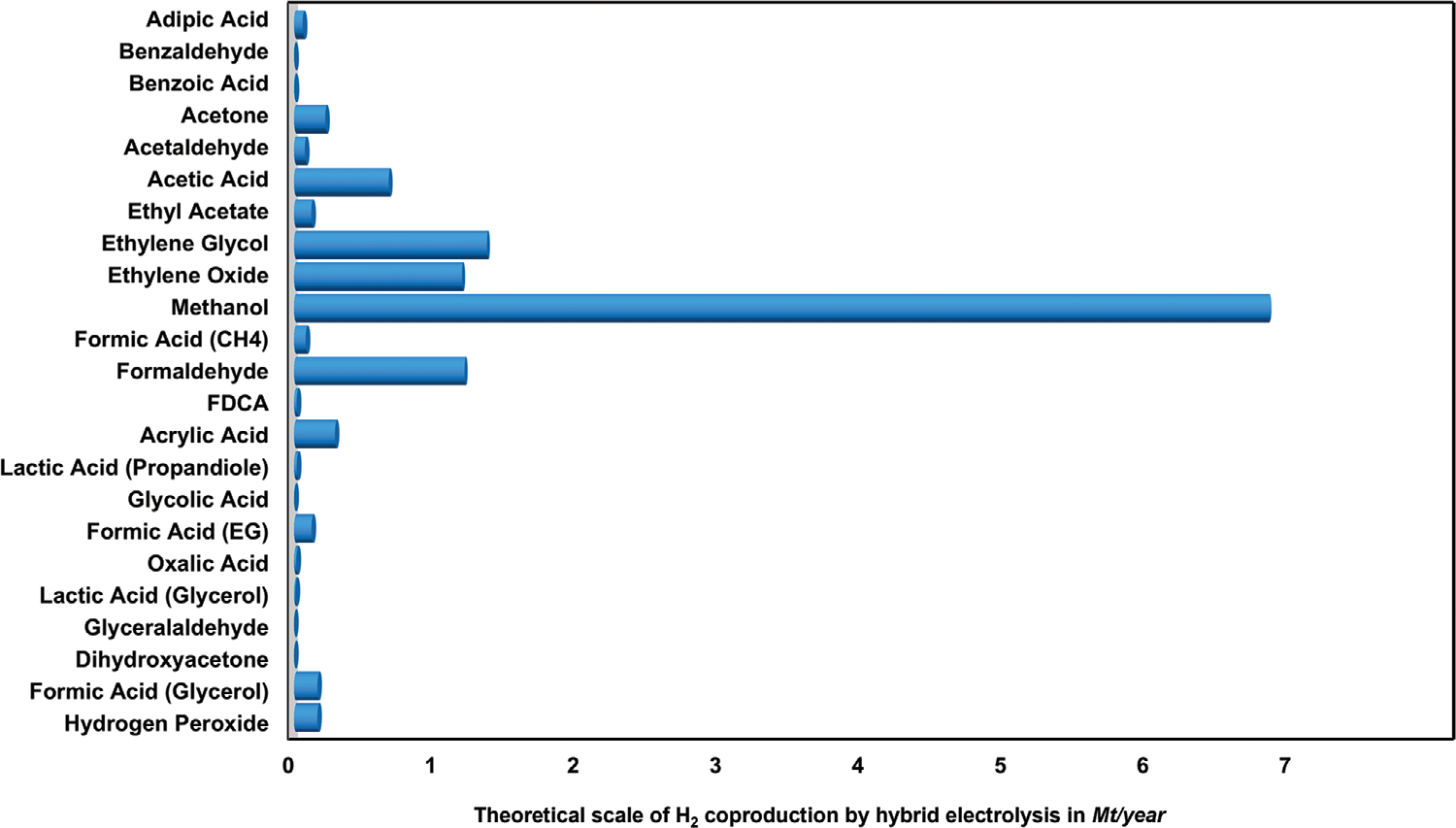
Hydrogen production potential of different hybrid water electrolysis processes by the demand of the anodically produced organic products. For this estimation, the annual production of the value‐added chemicals from Table 2 was converted to mol and multiplied by the ratio of H_2_ molecules produced per molecule of anodic product. Subsequently, the molar annual production potential of H_2_ was converted back to Mt. If the product of the hybrid water electrolysis is the same for different processes, the feedstock is specified in brackets.

**Figure 3:**
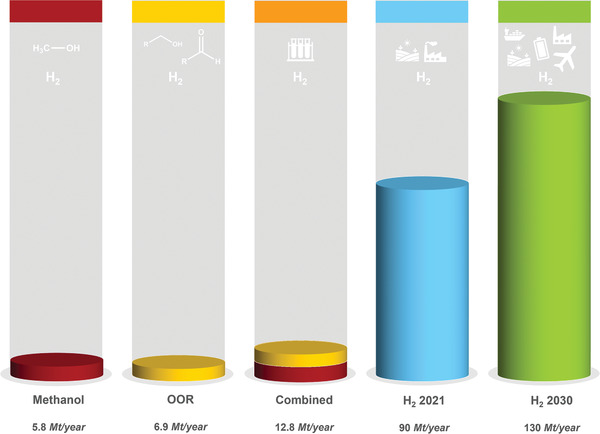
Scale of hydrogen production through hybrid water electrolysis compared to the global hydrogen demand. Methanol is currently produced thermocatalytically from syngas (H_2_ and CO). Therefore, its production from methane would not only produce green hydrogen, but also prevent the H_2_ consumption of the methanol synthesis. OOR (organic oxidation reactions) is the potential scale of H_2_ production assuming that the current demand for all chemicals in Figure 2 is produced by hybrid water electrolysis. Data for the hydrogen demand is taken from the Global Hydrogen Report of the International Energy Agency 2022.^[^ ^38^ ^]^

In order to estimate the market potential of organic oxidation and oxygenation reactions in hybrid water electrolysis, the annual demand for oxidation products should be compared considering the electrons provided per mol of oxidation substrates and not the annual production in gravimetric mass. In this regard, hydrogen requires one electron per proton (molecular weight 1 g mol^−1^), while organic feedstocks and products are often magnitudes heavier but supply only 2–8 electrons. For a simple estimation of the potential scale of hybrid water electrolysis, we assumed all the value‐added products to be solely produced by electrooxidation to calculate how much hydrogen could be produced with the obtained electrons and protons. **Figures** **2** and **3** show that no alternative oxidation product can match the scale of the current hydrogen demand. For example, the annual production of ethylene glycol is 24 Mt. However, its molecular weight is 62 g mol^−1^, while only two electrons are provided during the oxidation of ethylene. Therefore, only 1.37 Mt of hydrogen can be produced with the process. The combined demand for anodic products would be able to supply 12.8 Mt of hydrogen at most, which accounts for 14% (2021) or 10% (2030) H_2_ production, respectively (Figure 3). The biggest contribution could come from electrocatalytic methane oxidation to methanol, a two‐electron process. This is unsurprising as the annual demand for methanol is 110 Mt and thus significantly larger than for the other feedstocks. Furthermore, methanol has a small molecular weight and thus provides many electrons per produced mass. All other oxidation products combined would only be able to supply 6.5% of the current annual global hydrogen demand.

Still, the valorization of ethylene to either ethylene oxide or ethylene glycol looks promising with a combined potential hydrogen generation of 4 Mt. In addition, the implementation of HMF and glycerol as platform chemicals is still in its infancy. FDCA is a promising candidate as an intermediate for sustainable polymers replacing PET, which has an annual production of 35 Mt.^[^ ^51^ ^]^

Besides the demand of the products, feedstock requirements exist for the industrial‐scale application of hybrid water electrolysis. A limiting factor are regional mismatches between renewable power production and feedstock production, as they are unevenly distributed, increasing the overall cost of the process due to the transportation of biomass. The overall environmental impact of the hybrid water splitting process must also be considered. In this regard, Luo et al. compared the global warming potential of bio‐derived methanol, glycerol, and ethanol feedstocks in hybrid water electrolysis with the steam reforming process. In all cases, they find substantially lower emissions for hybrid water electrolysis. However, also hybrid water electrolysis can lead to carbon dioxide emissions.^[^ ^12^ ^]^ The main factor influencing the carbon footprint of hybrid water electrolysis is the production/sourcing of feedstocks and nonrecovered electrolyte additives (see next paragraph). Traditional water splitting powered by renewable energy sources is free of carbon dioxide emissions. This underlines that organic oxidation reactions also inherit a higher global warming potential, even though they are more energy efficient and might generate additional revenue through the value‐added products. While HMF or glycerol are biomass‐based substrates, most other chemicals in Table 2 are mainly derived from crude oil. In perspective, even for these petrochemically derived substrates, hybrid water splitting systems can be nearly emission‐free if low carbon footprint bio‐derived alternatives are developed.

Another crucial aspect is electrolyte purification. In contrast to the OER, the value‐added products of anodic organic oxidations remain in the electrolyte. Thus, to obtain the desired organic products, the electrolyte must be purified. This electrolyte purification step is a significant cost factor that strongly depends on the organic oxidation reaction. Furthermore, not only the organic product must be recovered but also electrolyte additions such as KOH.^[^ ^12^ ^]^ Otherwise, KOH must be added to the electrolyzer continuously, which is a substantial cost factor, and, depending on the KOH production, a cause of carbon dioxide emissions.^[^ ^12^ ^]^ This is not the case for the OER, as the KOH can remain in the electrolyzer and only high‐purity water is added.^[^ ^8^ ^]^ Therefore, it is crucial to develop desalination procedures to regain the potassium hydroxide. In this regard, Sirisangsawang et al. have demonstrated the conversion of sodium lactate to lactic acid and sodium hydroxide using a cation exchange membrane electrolytic cell.^[^ ^52^ ^]^ Nevertheless, these purification steps further complicate hybrid water electrolysis systems compared to traditional water splitting and are a substantial cost factor.

### 3.3 Selectivity, Activity, and Stability in Hybrid Water Electrolysis

Replacing the OER with alternative organic oxidation reactions brings multiple new challenges for the electrochemical system. The reaction mechanism, catalytical steps, and optimal reaction conditions differ significantly depending on the substrate and desired product. Therefore, resolving the mechanisms and developing novel and earth‐abundant catalysts capable of selectively oxidizing feedstocks into a single desired product at industrially relevant current densities on an extended time scale is crucial. Furthermore, operation conditions (pH, temperature, cell design) must also be tuned for each reaction. A few selected examples are discussed in this section to give an impression of these factors.

Just recently, Wang et al. have conducted an extensive screening of organic substrates for an improved Ni(Fe)OOH electrode, including various alcohols, aldehydes, ketone, and amines achieving current densities of industrial relevance as well as high selectivity of 80%–90% for all substrates. However, most alcohols were oxidized to formic acid, including glycerol, preventing the formation of potentially interesting partially oxidized products such as lactic acid or dihydroxyacetone.^[^ ^53^ ^]^ While this study is interesting for comparing the overpotential and current densities of different organic oxidations with the same catalyst, it gives little insight into pathways to divergent products of the same feedstock.

Glycerol is an excellent example concerning the selectivity challenge in hybrid water electrolysis, as it has at least ten different thinkable oxidation products.^[^ ^54^ ^]^ In this regard, McKenna et al. have used a NiOOH‐based catalyst at different pH and potential ranges to examine the influence of the operation conditions. They found that at high pH (>13),^[^ ^30^ ^]^ formic acid was the dominant product, while lowering the pH (9) would shift the selectivity towards secondary alcohol oxidation to dihydroxyacetone, thereby preventing C—C cleavage. In addition, they proposed two different dehydrogenation mechanisms for NiOOH: i) a potential independent hydrogen atom transfer regenerating the oxidant and ii) a potential dependent mechanism. The latter is responsible for the C—C cleavage with growing positive potential.^[^ ^55^ ^]^ Pei et al. observed that the glycerol concentration in the electrolyte during anodic oxidation influences the system's performance as the electrolyte's resistance increased over 0.2 mol L^−1^, due to the large viscosity and the associated weakened convective mass transfer.^[^ ^56^ ^]^ These findings underline the complexity and multitude of influential factors when tuning the selectivity and activity toward a desired product. The potential, substrate concentration, and catalyst must be coordinated to achieve a successful conversion.

Regarding the reaction media, hybrid water electrolysis has been mostly conducted in aqueous alkaline media, as the earth‐abundant and relatively cheap 3d transition metals dissolve in acidic to neutral media. Thus, precious metal catalysts like platinum, palladium, or gold are required, undermining the process's cost competitiveness and industrial‐scale application as discussed earlier. Thus, developing nonprecious metal catalysts that are stable in neutral and acidic conditions could open new paths to other oxidation reactions unavailable in alkaline conditions. For a recent investigation on the various factors influencing the stability and activity of non‐noble metal catalysts in alkaline media see ref.^[^ ^9^ ^]^ In addition, some organic substrates are not soluble in water, for example, larger hydrocarbons like cyclooctene make water an unsuitable electrolyte. To tackle this issue, organic solvents can be mixed with water to increase substrate solubility. Acetonitrile received considerable attention as an electrolyte in lithium ion batteries due to its oxidative stability.^[^ ^57^ ^]^ This characteristic can also be utilized when overoxidation should be avoided. On this matter, Kyoungsuk et al. demonstrated the electrochemical epoxidation of cyclooctene in a mixture of acetonitrile and water. By utilizing a Mn_3_O_4_‐based nanocatalyst, they achieved 30% selectivity toward cyclooctene oxide, a product potentially suffering from overoxidation.^[^ ^58^ ^]^ While cyclooctene is a good choice as a model compound, the small epoxides, first and foremost oxirane, are of higher interest considering our market scope analysis findings (Table 2). The feedstock for such a conversion would be ethylene, which could also be converted to ethylene glycol. To the best of our knowledge, both reactions have only been realized electrochemically using noble metals (Pd/Ru) and chlorine redox mediators, increasing the system's complexity and complicating purification.^[^ ^59^, ^60^, ^61^ ^]^

While the efforts outlined above are interesting and important, they also emphasize the premature status quo of hybrid water electrolysis. Selectivity remains a challenge, and there is a substantial need for systematic research regarding the structure of the active sites and oxidation states of the metal catalysts through in‐situ studies to gain a fundamental understanding of these multistep electron transfer reactions. Only then it will be possible to boost the electronic properties comparable to approaches used in OER, but tailored for a specific organic oxidation.^[^ ^62^ ^]^ Long‐term stability remains another unsolved issue that must be addressed. Until now, no alternative oxidation reaction was tested on an extended time scale (days to weeks) under relevant conditions, so little is known about the potential poisoning of the electrodes by the organic compounds (carbonate or coke formation) or catalyst degradation.^[^ ^12^ ^]^ Currently, the hybrid water electrolysis community mainly focuses on the conversion of a single batch, in such a way that the organic substrate is once added to the electrolyte and then the process is stopped after full conversion (usually in the range of a few hours). A continuous operation setup must be applied to test the long term‐stability in a meaningful way, where the electrolyte is replaced multiple times or a flow cell is constructed.^[^ ^63^ ^]^ In such systems, academic research should aim for stability tests in the range of weeks to months to check the suitability of their catalysts for real‐world applications.

## 4 Conclusion

Hybrid water electrolysis has the potential to produce low‐emission hydrogen simultaneously with value‐added chemicals. By substituting the OER with alternative oxidation reactions, energy and atom efficiency are potentially enhanced. Although the concept is promising to make a meaningful contribution to the decarbonization of our society, substantial challenges remain:

- It is true that the OER has sluggish kinetics and requires large overpotentials in addition to the already high potential of 1.23 V_SHE._ However, most organic oxidation reactions currently suffer from even higher kinetic overpotentials. To overcome these, novel electrocatalysts must be developed from earth‐abundant materials specifically tailored for organic oxidation reactions.
- The scale of hybrid water electrolysis is limited. It can be estimated from the product demand and compared to the global hydrogen production. In this regard, for the most promising, potential industrial‐scale, anodic oxidation reactions, we calculated that they would account for only 14% of the current global hydrogen production, even if the worldwide demand was supplied by hybrid water electrolysis. In this regard, it is crucial to consider that the hydrogen demand is expected to increase enormously within the next decades and that carbon dioxide reduction also requires protons and electrons from the OER or its alternative processes.
- When screening for suitable alternatives for the OER, techno‐economic factors are also crucial. In this regard, feedstock cost, availability, and carbon footprint, together with the selling price and demand of the value‐added products, are key parameters discussed herein.
- Electrolyte purification is another significant challenge. In traditional water splitting, the electrolyte can remain in the electrolyzer and only high‐purity water is added. In hybrid water electrolysis, purification and separation of the anodic products from the electrolyte and recovery of electrolyte additives, such as KOH, are required, which can be challenging and cost‐intensive.
- Several challenges regarding the stability and selectivity of the system remain. Hybrid water electrolysis is a highly complex system, in which all parameters can significantly change the outcome of the oxidation. Understanding the reaction mechanism and the nature of active sites is crucial for developing novel catalysts that selectively oxidize a substrate. Regardless of the oxidation reaction, long‐term stability remains unresolved.

Academic research on hybrid water electrolysis is thriving. However, many challenges remain for its industrial‐scale application. We anticipate that this perspective provides an overview of these challenges and guides the academic research community toward solving industrially relevant research questions.

## Conflict of Interest

The authors declare no conflict of interest.
